# A mouse protein that localizes to acrosome and sperm tail is regulated by Y-chromosome

**DOI:** 10.1186/1471-2121-14-50

**Published:** 2013-11-20

**Authors:** Rupa Bhattacharya, Manju S Devi, Vishnu M Dhople, Rachel A Jesudasan

**Affiliations:** 1 Centre for Cellular and Molecular Biology, Hyderabad, Andhra Pradesh, India; 2 Present Address: Lawrenceville, New Jersey 08648, USA; 3 Department of Functional Genomics, Interfaculty Institute for Genetics and Functional Genomics, Universitätsmedizin Greifswald, Friedrich-Ludwig-Jahn-Straße 15 a, Greifswald 17487, Germany

**Keywords:** MAST, Acrosome, Sperm tail, Co-IP, Yq-del mice

## Abstract

**Background:**

Acrosomal proteins play crucial roles in the physiology of fertilization. Identification of proteins localizing to the acrosome is fundamental to the understanding of its contribution to fertilization. Novel proteins are still being reported from acrosome. In order to capture yet unreported proteins localizing to acrosome in particular and sperm in general, 2D-PAGE and mass spectrometry analysis of mouse sperm proteins was done.

**Results:**

One of the protein spots identified in the above study was reported in the NCBI database as a hypothetical protein from Riken cDNA 1700026L06 that localizes to chromosome number 2. Immunofluorescence studies using the antibody raised in rabbit against the recombinant protein showed that it localized to mouse acrosome and sperm tail. Based on the localization of this protein, it has been named *m*ouse *a*crosome and *s*perm *t*ail protein (MAST, [Q7TPM5 (http://www.ncbi.nlm.nih.gov/protein/Q7TPM5)]). This protein shows 96% identity to the rat spermatid specific protein RSB66. Western blotting showed that MAST is expressed testis-specifically. Co-immunoprecipitation studies using the MAST antibody identified two calcium-binding proteins, caldendrin and calreticulin as interacting partners of MAST. Caldendrin and calreticulin genes localize to mouse chromosomes 5 and 8 respectively. In a Yq-deletion mutant mouse, that is subfertile and has a deletion of 2/3rd of the long arm of the Y chromosome, MAST failed to localize to the acrosome. Western blot analysis however, revealed equal expression of MAST in the testes of wild type and mutant mice. The acrosomal calcium-binding proteins present in the MAST IP-complex were upregulated in sperms of Yq-del mice.

**Conclusions:**

We have identified a mouse acrosomal protein, MAST, that is expressed testis specifically. MAST does not contain any known motifs for protein interactions; yet it complexes with calcium-binding proteins localizing to the acrosome. The misexpression of all the proteins identified in a complex in the Yq-del mice invokes the hypothesis of a putative pathway regulated by the Y chromosome. The role of Y chromosome in the regulation of this complex is however not clear from the current study.

## Background

The male germ cell, sperm is responsible for delivering paternal genome to the female counterpart, the oocyte during fertilization. Spermatogenesis, the process of development of sperm from undifferentiated germ cells is accomplished through a series of cellular differentiation processes, which require a fine-tuned milieu that regulates the expression of genes and proteins [1-4]. Paternal genome is transferred to the egg cell, during the fertilization, a process mediated by the acrosome reaction. The acrosome is a lysosome-like membrane-bound organelle covering the head of spermatozoon. Acrosome formation is a crucial step in spermatogenesis. In mammals, acrosome biogenesis begins in the late spermatocyte and continues throughout the first half of spermiogenesis. In addition to well-known proteins involved in fertilization, enzymes (proteases, glycosidases, esterases, acid phosphatases, aryl sulfatases), antigens, and various ZP-binding proteins [5], acrosome contains some less-characterized proteins grouped as sperm acrosome associated (SPACA) family proteins. This family has been created based on the cellular localization of its members, but the functions of some of these proteins are still not known [6].

As the development of sperm specialized for fertilization is a unique process that occurs only in testis, gaining an understanding of spermatogenesis and fertilization requires identification and characterization of proteins expressed in testicular germ cells. Although protein profiles of spermatogonia, sperm, and different developmental stages of the testes have been studied in different mammalian species, the proteins involved in spermiogenesis are still not fully characterized.

Approximately 2300 genes (4% of the mouse genome) are transcribed only in male germ cells [7]. Many of these genes encode proteins involved in acrosome formation, sperm–egg interactions, and fertilization; yet a large fraction of them remains uncharacterized. The advent of 2D PAGE and mass spectrometry has provided the benefit of high throughput analysis of spermatozoa at the molecular level [8]. In 1997, Naaby-Hansen and colleagues [9] established a database of 1397 protein spots from human sperm. In 2007, Li et al. [10] identified 3872 different protein spots from a high-resolution 2D PAGE map from human spermatozoa.

A novel protein SPACA7 that localizes to the acrosome was described in 2012 by Korfanty and colleagues [6]. Thus new proteins localizing to acrosome are still being discovered. Therefore, we performed sperm proteome analysis using 2D PAGE and mass-spectrometry to identify novel proteins in mouse sperm. One of the spots chosen for analysis from a 2D-gel in the pI range of 5–8 was reported as hypothetical protein from Riken cDNA 1700026L06 localizing to mouse chromosome number 2. Immunolocalization studies using antibody raised to the protein in the lab showed localization of the protein to acrosome and sperm tail. The protein has been named MAST [Q7TPM5 (http://www.ncbi.nlm.nih.gov/protein/Q7TPM5)] based on its localization on the sperm. Multi-tissue western blot analysis showed that this is a protein that is specific to mouse testis. Studies using Co-IP showed that although this protein that has no known motifs, interacts with two calcium-binding proteins calreticulin and caldendrin localizing to the acrosome. Comparative western blotting using the wild type and a subfertile Yq-deletion mutant mouse showed that this protein is absent in Yq-del sperm regardless of the fact that this gene localizes to an autosome.

## Results

### Identification of hypothetical protein

**Figure 1:**
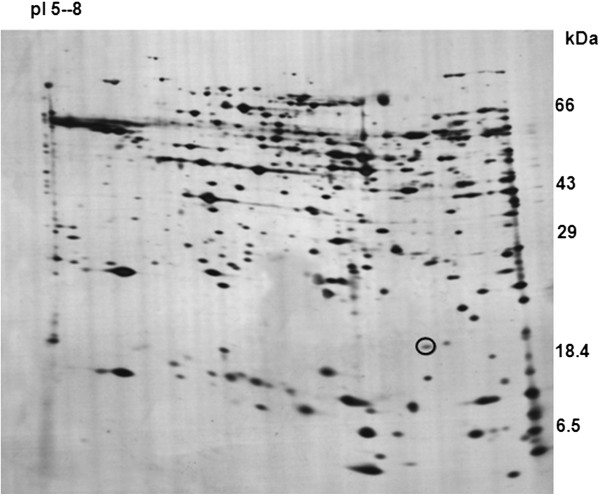
**Identification of a mouse sperm protein.** Figure shows representative Coomassie blue stained 2D PAGE of mouse sperm proteome in the pI range of 5–8. The protein spot identified in the molecular range of 20 kDa is encircled.

Total proteome from specialized cells such as spermatozoa are good source of identifying unreported proteins that may have functional significance associated with the cell or the tissue of origin. Protein spots isolated from 2D gel of mouse sperm proteome in the pI range of 5–8 were subjected to in-gel digest and MALDI-TOF analysis. The mass fingerprint obtained was submitted to probability-based search engine MASCOT (http://www.matrixscience.com). One of the spots encircled in Figure 1 was identified as a hypothetical protein (1700026L06 Riken cDNA) with high MOWSE score by MASCOT. Identity of this protein was further established by de-novo sequencing using MS-MS. Analysis of the spectra yielded an MS tag of ten amino acids – VSQSNDLSSL - (Additional file 1: Figure S1). BLAST against the NCBI database revealed this as a hypothetical protein with only the Riken cDNA 1700026L06, localizing to mouse chromosome number 2. The position of the protein spot on 2D gel matched the expected pI and molecular weight of the hypothetical protein from the Riken cDNA 1700026L06. The MS tag obtained is highlighted in the protein sequence as shown below:

MyHits motif scan did not identify any known motifs in MAST (http://myhits.isb-sib.ch/cgi-bin/motif_scan) [11].

**Figure 2:**
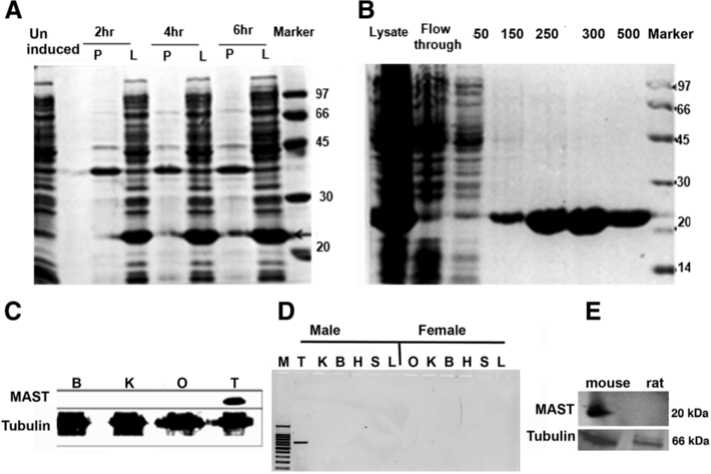
**Expression profiling of MAST. (A)** Shows the protein profile of cultures induced at 2 h, 4 h and 6 h after induction with IPTG. The lysates (L) show overexpressed protein from 2 h onwards (indicated by arrow). Corresponding pellets (P) did not contain the overexpressed protein. Lysate from uninduced cell served as negative control. **(B)** Panel B shows the profile of the proteins eluted using a Ni-NTA column at 50, 150, 250, 300 and 500 mM concentrations of imidazole. Pure protein out in 300 and 500 mM imidazole. Lysate and the flow through fractions were loaded as controls. **(C)** Multi-tissue western blotting with antibody raised against MAST protein using extracts from mouse brain, kidney, ovary and testis shows a signal only in testis, indicating testis specific expression. Tubulin antibody was used as loading control. **(D)** Semi quantitative RT PCR using 6 tissues (kidney, brain, heart, spleen, liver from males and females, testis and ovary) shows a signal only in testis confirming the male restricted and testis specific expression of the MAST transcript. **(E)** Western blot analysis using testicular lysates from mouse and rat with the MAST antibody showed expression in mouse, not in rats.

As we identified a hypothetical protein for which the cDNA sequence was available in the database, in order to characterize the protein the cDNA was cloned in pET-15b vector, over-expressed in BL21 (DE3) cells (Figure 2A), and purified on a Ni-NTA column (Figure 2B). MALDI analysis confirmed that the overexpressed protein corresponded to Riken cDNA 1700026L06.

### Tissue specific expression and immunolocalization of a mouse protein

*In vivo* expression of the newly identified protein was checked on a multi tissue western blot using the primary antibody raised in the laboratory. Presence of a single band of about 20 kDa only in testis indicated mouse male restricted expression of the protein (Figure 2C). Semi quantitative RT-PCR using mouse tissues kidney, brain, heart, spleen, liver from both males and females, testis and ovary showed testis-specific expression of MAST transcript (Figure 2D). This confirmed the male specific and testis-specific expression of the protein. RSB66 is reported as the rat homologue of LOC1700026L06 (http://www.ncbi.nlm.nih.gov/nuccore/NM_181694.2). Western blot analysis using the polyclonal antiserum raised against MAST did not identify a signal in rat sperm lysate indicating absence of cross-reactivity from mouse to rat (Figure 2E).

**Figure 3:**
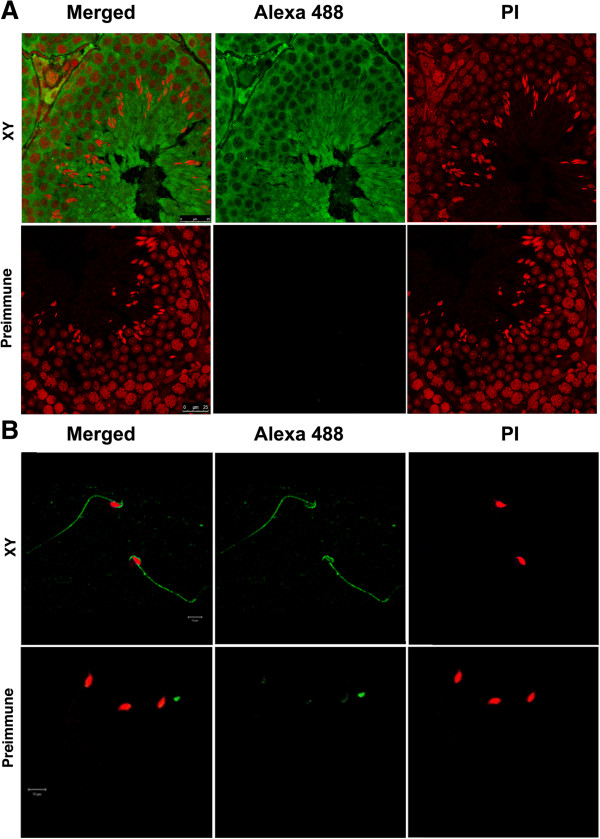
**Immunolocalization of MAST on testis and sperms of normal males. (A)** Immunolocalization of MAST protein onto testicular sections using indirect immunofluorescence. Localization of MAST antibody was detected by rabbit polyclonal secondary antibody conjugated with Alexa fluor 488. Propidium Iodide (PI) was used as the counterstain. Merging of the Alexa and PI captures on a confocal microscope shows cytoplasmic localization of the protein in all the cell types in testis (XY). Intense staining is observed in elongated spermatids present towards the lumen of the seminiferous tubules. Pre-immune serum was used as the negative control; magnification was 100×; scale bar = 25 μm. **(B)** Immunolocalization on sperm: Immunolocalization with antibody to MAST protein showed that in sperms the protein localized to the sperm head with acrosomes showing intense staining. Protein was also present on sperm tails. DAPI is used as the counterstain for nucleus and pseudo color red is used for representation. Magnification- 63×; scale bar = 10 μm.

Immunolocalization on testis sections of the wild type RIII strain of mice showed cytoplasmic localization (Figure 3A). The protein was expressed from almost all the cell types of testis, with abundant expression in round and elongated spermatids. Immunostaining of caudal sperms showed localization of the protein onto sperm head, with intense staining at the acrosome region. Protein was also present on midpiece and principle piece of sperm tails (Figure 3B). Based on the localization of the protein on to the acrosome and sperm tail, it has been named the mouse acrosome sperm tail (MAST) protein. We have submitted the protein to the NCBI database and it has now been given the accession number Q7TPM5 (http://www.ncbi.nlm.nih.gov/protein/Q7TPM5).

### Interaction with calcium binding proteins caldendrin and calreticulin

**Figure 4:**
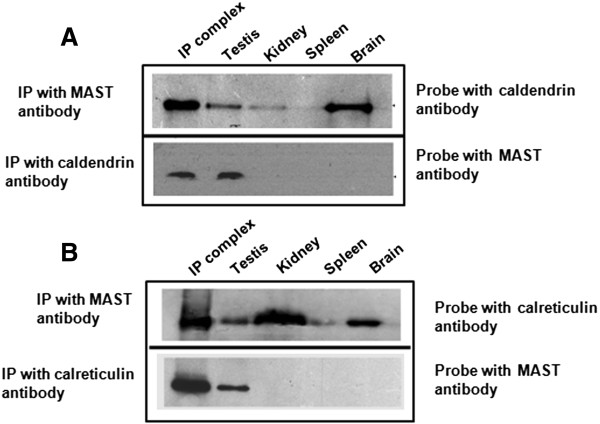
**Co-immunoprecipitation and Western blot analysis. (A)** The upper panel shows the presence of caldendrin in IP complex of MAST antisera on probing with antisera raised to caldendrin. Signals of the same molecular weight are also present in the lysates (without immunoprecipitation) from mouse testis, kidney and brain indicating the presence of caldendrin in these tissues as well. In the lower panel IP with caldendrin antisera that is probed with MAST antisera shows the presence of MAST protein in the IP complex and testis lysate only. MAST is not present in other tissues such as kidney, spleen and brain. **(B)** Co-immunoprecipitation of MAST and calreticulin: Antisera of MAST used for IP and probed with calreticulin antibody shows the presence of calreticulin protein in the IP complex and the lysates from testis, kidney spleen and brain, with high expression in kidney. Calreticulin antibody used for immunoprecipitation and probed with antisera of MAST protein shows the presence of MAST protein in the IP complex and testis lysate only.

Localization of MAST to the acrosome indicated possible functions in acrosome reaction/fertilization. In order to query the physiological role of MAST, interaction with other proteins localizing to the acrosome was studied by immunoprecipitation studies. Three acrosomal proteins considered in this study were caldendrin, acrosin and calreticulin. First, caldendrin was probed for interaction with MAST, if any. The immunopulldown (IP) using MAST antibody when probed with caldendrin antibody raised in the laboratory identified a signal on a western blot. When the pull down product of caldendrin antibody was probed with the MAST antibody also, a signal was identified that corresponded to the molecular weight of MAST from mouse testis and the overexpressed recombinant protein. Thus the Co-IP studies confirmed the interaction between caldendrin and MAST (Figure 4A). Co-IP studies using antibodies to calreticulin and MAST showed the presence of calreticulin in the pull down complex of MAST and vice versa; i.e. MAST antibody identified a signal on the western blot containing the complex pulled down with calreticulin antibody (Figure 4B). As caldendrin and calreticulin were both identified in the pulldown complex of MAST, this showed the presence of the three proteins in the same complex. Co-IP studies using MAST and acrosin antibodies showed that they did not interact (result not shown). These results show interaction of MAST with the calcium binding proteins caldendrin and calreticulin, but not with acrosin. Interaction of MAST was also tested with the testis-specific superoxide dismutase (SOD) that localizes on the mouse sperm tail as MAST. Co-IP using antibodies to MAST and SOD did not yield any signal (result not shown). This further confirms the interaction of MAST with the two calcium-binding proteins. The gene corresponding to caldendrin localizes to chromosome number 5 and that for calreticulin to chromosome number 8 in the mouse.

### Comparative studies using a Yq-deleted strain of mouse

**Figure 5:**
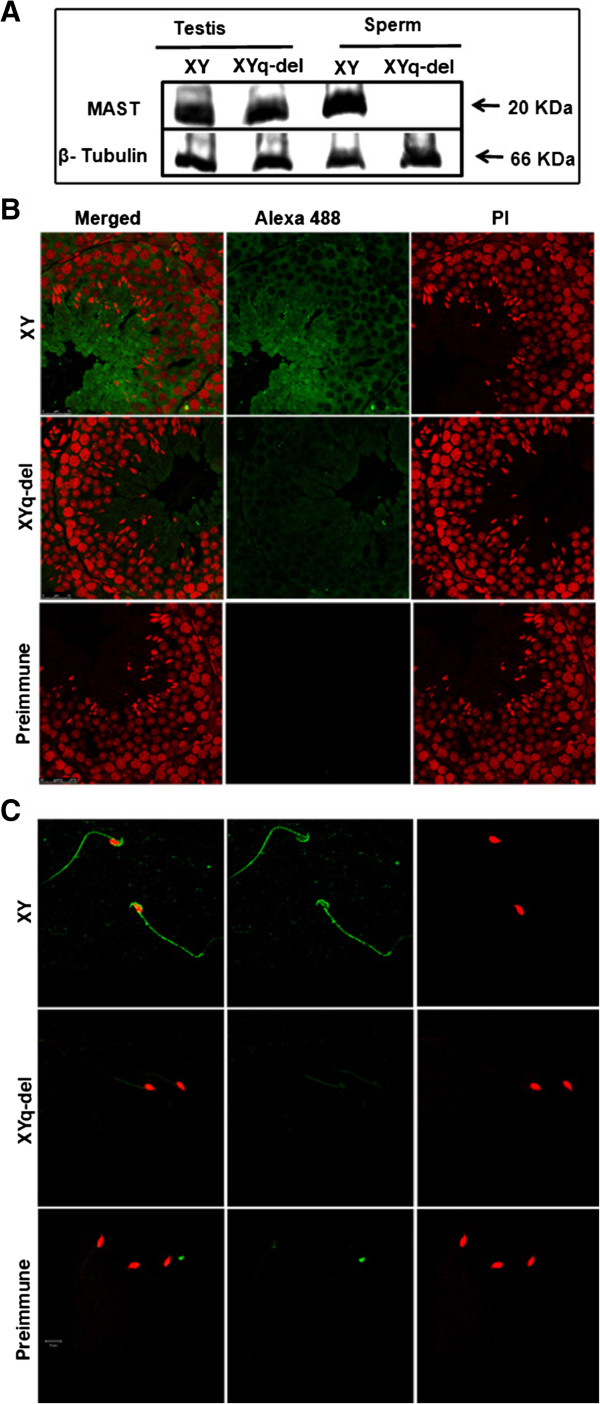
**Comparative analyses of normal and subfertile mice using MAST antibody. (A)** Comparative Western blotting with testes and sperm lysates from normal (XY) and subfertile (XYq-del) mice probed with MAST antibody shows presence of a 20 kDa protein in comparable quantities in the testis lysates of both. MAST was absent in the subfertile sperm proteome. Tubulin antibody served as the loading control. **(B)** Shows cytoplasmic localization of the protein in the entire cell types of normal (XY) testis. In the subfertile (Yq-del) testis there is a substantial reduction in signal, indicating either reduced expression or alternatively lack of localization to the correct locus. Pre-immune serum was used as negative control and PI was the counterstain. Magnification - 100× and scale bar = 25 μm. **(C)** Comparative immunolocalization in sperms: Immunolocalization with antibody to MAST protein showed that in normal (XY) sperms the protein localizes to the acrosome and sperm tail. In sperms from subfertile (XYq-del) mice a very faint signal is observed only in the midpiece of sperm tail. DAPI was used as the counterstain for nucleus and pseudo color red was used for representation. Magnification - 63×; scale bar = 10 μm.

A strain of mutant mouse wherein there is a 2/3rd deletion of the heterochromatic long arm of the Y chromosome is deleted shows sperm abnormalities and subfertility [12]. Comparative immunoblotting using testis and sperm lysates from normal and Yq-del mice showed that MAST was present in testes of normal and Yq-del mice in equal quantities; MAST was present in sperm lysate from wild type mice, but absent in sperms from Yq-del mice (Figure 5A). Comparative immunolocalization studies on testes sections of wild type and Yq-del mice showed that in Yq–del testes signal was barely present in cells of the basal lamina and round spermatids and was substantially reduced in elongated spermatids (Figure 5B). Yq-del sperms showed only a faint signal restricted to midpiece (Figure 5C). Thus MAST was found to be absent in Yq-del sperms by western blot analysis and immunolocalization. Western blot using normal and Yq-del testes lysates showed equal expression of MAST, but immunolocalization onto Yq-del testis sections showed a drastic reduction in signal in comparison to that of the wild type. As there was no difference in the intensity of the signal between the normal and Yq-del mice by western blot analysis, these results indicate that there is no reduction in expression of MAST in Yq-del testis; the reduction in intensity in immunolocalization studies might reflect failure of localization to the correct locus rather than a quantitative difference in expression.

### Upregulation of caldendrin and calreticulin in Yq-del mice

**Figure 6:**
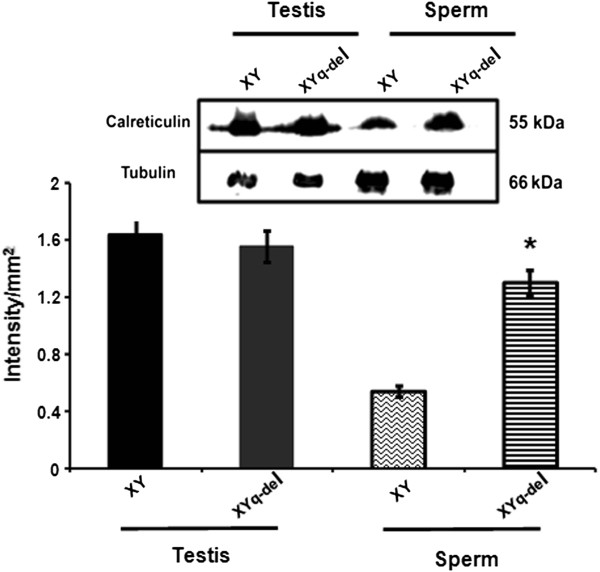
**Comparative expressions of calreticulin in normal and subfertile mice by Western blotting.** The panels show comparative expression of calreticulin (55 kDa) in testes of normal (XY) and subfertile (XYq-del) mice. Calreticulin is over expressed in the sperms from the subfertile mice. β-Tubulin is the loading control. The intensities of the bands were quantitated using Gene tool software and normalised against tubulin. *indicates statistical significance when compared to XY sperm.

**Figure 7:**
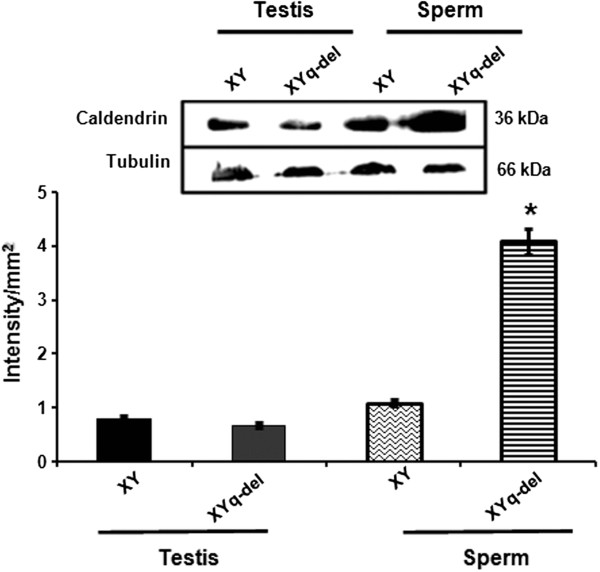
**Comparative expressions of caldendrin in normal and subfertile mice by Western blotting.** The panels show comparative expression of caldendrin (36 kDa) in testes of normal (XY) and subfertile (XYq-del) mice. Caldendrin is over expressed in the sperms from the subfertile mice. β-Tubulin is the loading control. The intensities of the bands were quantitated using Gene tool software and normalised against tubulin. *indicates statistical significance when compared to XY sperm.

The expression of the interacting partners of MAST, caldendrin and calreticulin were also studied in Yq-del sperm by western blot analysis. Immunoblotting using anti calreticulin antibody on sperm lysates from normal and Yq-del males showed 2.6 fold higher expression in Yq-del sperms (Figure 6). The expression of caldendrin, the other interacting protein was found to be 4 fold higher in Yq-del sperms when compared to normal sperms (Figure 7).

## Discussion

Sperm bears the responsibility of delivering the male genome to its female counterpart, and thus has specialized function of motility in male and female reproductive tracts, recognition of female gamete and fusion thereafter. Sperm proteins have a major role in sperm function and thereby male fertility [13]. In the current study we have identified a protein that was reported in the NCBI database as a hypothetical protein, from Riken cDNA 1700026L06 with homology to rat RSB66 and human C9ORF9 proteins (http://www.ncbi.nlm.nih.gov/protein/Q7TPM5). Since the identity of the protein was established by MALDI and MS-MS based peptide mass fingerprinting, this study provides evidence and confirms for the first time that the protein encoded by 1700026L06 Riken cDNA does exist *in vivo*. RSB66 on the other hand localizes only to spermatids in rats [14] although it shows 96% identity to the mouse protein. Multi-tissue immunoblotting using antibody raised against the protein identified it only in mouse testis thereby confirming the male-specific expression of the protein. In mature epididymal spermatozoa protein localizes to the sperm acrosome and is present in midpiece and principle piece of sperm tail. Based on the presence in mouse acrosome and sperm tail the protein has been named as mouse acrosome and sperm tail ‘MAST’ protein.

Localization to the acrosome would suggest a role in either recognition or penetration of the female gamete. The absence of known functional motifs in MAST did not enable prediction of functions. In the absence of signal sequences it is not clear as to how the protein localizes to the acrosome. In order to infer any pathway that the protein may be involved in, we attempted to identify the interacting partners. As MAST localizes to the acrosome and sperm tail, proteins localizing to sperm acrosome and tail such as acrosin, caldendrin calreticulin and superoxide dismutase were chosen for the study. Co-IP studies showed that MAST complexes with two proteins calreticulin and caldendrin both of which localize to the acrosome and bind calcium.

Calreticulin is a calcium-binding acrosomal protein implicated in sperm functions such as motility, capacitation and acrosome reaction [15,16]. Calreticulin localizes also to the neck and midpiece of the human sperm [17]. It has many cellular functions including chaperon activity [18,19]. Whether the over-expression of a calcium binding chaperon, calreticulin has any role in non-localization of MAST is not clear from the present study.

Caldendrin is expressed abundantly in the somatodendritic compartment of principal neurons in brain regions with a laminar organization like cortex or hippocampus [20,21]. Caldendrin, which is presumed to be a stimulus dependent multifunctional regulator of intracellular calcium ions also localizes to the rat acrosome [22]. Brain and testis are known to show inexplicable similarity in gene expression [23]. This study is however, the first report to show interaction of caldendrin with the mouse testis-specific protein MAST. Due to the presence of minimal cytoplasm and limited organelles, ions such as Ca^2+^ play pivotal role in manifestation of cellular response of sperm [24]. It is possible that MAST has Ca^2+^ dependent cellular function(s) such as calcium signaling and/or exocytosis associated with acrosomal reaction.

It is interesting to note that all the three proteins identified in a complex are misexpressed in sperms of the Yq-deleted mutant mice. The significance of over-expression of caldendrin and calreticulin, the interacting partners of MAST in sperms of a strain of subfertile mouse is not evident from the present study. As MAST does not localize to sperms in Yq-deleted subfertile mice, these proteins may play a role in the localization of MAST to its functional locus, the acrosome. Earlier studies from the lab reported putative regulation of CDC2L2 by a Y-heterochromatin derived noncoding RNA testis-specifically in human [25]. The deregulation of proteins of a complex localizing to the acrosome in Yq-deleted mice, perhaps indicates the existence of a pathway regulated by the Y heterochromatin, the region deleted in the mutant mouse. The report of subfertility in Yq-del mice supports the above hypothesis [12]. Further studies are required to unravel this pathway. However based on the partnership between MAST and these calcium-binding proteins, all of which are deregulated in subfertile mice, a pivotal role in sperm function can be envisaged. These proteins might have roles in acrosome reaction/fertilization, as all these proteins localize to the acrosome in normal mice.

## Conclusions

This study identified a mouse sperm protein, that localizes to the acrosome and sperm tail. Although MAST does not contain any known motifs, it complexes with calcium binding proteins, which localize to the acrosome. Therefore further study of MAST would reveal novel motif(s) for interaction with calcium binding proteins. Results from the study also indicate the existence of a complex regulated by the Y chromosome. Detailed study of the proteins in the MAST complex would most likely unravel a pathway in mouse testis regulated by the Y chromosome as well.

## Methods

### Animals

The animal system used in this study is mouse (*Mus musculus*). The strains used were, RIII (XYRIII) and mutant RIII Yq-del (XY^RIII^qdel*)*. XY^RIII^q-del mutant strain of mice was a kind gift from Prof. Paul S. Burgoyne (MRC, UK). Antibodies to MAST and caldendrin were raised in young female rabbits. All the animals used in the experiments were bred and reared in the animal house of CCMB, in accordance with the guidelines from Indian Science Academy under CPCSEA (Committee for the Purpose of Control and Supervision of Experimental Animals). This study was approved by the Institutional Animal Ethics Committee (IAEC 65/28/2006).

### Tissue collection and isolation of sperms

Adult males of approximately 3 months of age were anaesthetized using chloroform and sacrificed by cervical dislocation. Cauda epididymis was dissected out and collected in PBS maintained at 37°C. Rest of the tissues like brain and testis were dissected out, rinsed in PBS and snap frozen in liquid nitrogen.

Dissected cauda epididymis was washed in warm PBS. In order to collect spermatozoa the epididymis was held tightly with forceps and was punctured with a needle. The spermatozoa were allowed to ooze out of the cauda, which was then collected into warm PBS and was allowed to stand for 10–15 minutes at 37°C in an air incubator so as to allow spermatozoa to disperse. The swim up fraction was collected carefully avoiding the epithelial cells and tissue debris at the bottom. A small part of the sperm fraction collected was checked under a light microscope to ensure that there was no contamination with other cells, such as epithelial, RBC and leucocytes. The sperm cells were then washed twice in PBS by spinning at 2000 rpm at 4°C for 10 minutes each.

### Two-dimensional gel electrophoresis (2D PAGE)

Testis and sperm lysates were prepared using cell lysis buffer (8 M Urea, CHAPS 4% (w/v), 40 mM Tris, Biolyte (3–10) 0.2% (w/v) and TBP (1 μl/100 μl). Depending upon the length of the immobilized pH gradient gel (IPG) strip 200 μg to 1 mg of protein was loaded. The first dimension separation of proteins based on pI (Isoelectric focusing, IEF) was performed in a 13 cm 5–8 IPG strip (pH 5–8 NL) Biorad overnight with passive rehydration and focusing program, as per manufacturer’s instructions. For the second dimension SDS-PAGE resolving polyacrylamide gel was prepared. Gradient gels of 8-20% were prepared by pouring equal volumes of two different concentrations of acrylamide solution to form a gradient with increasing concentrations of acrylamide from top to bottom using Amersham gradient former. Stacking gel was of 5% polyacrylamide. Prior to loading on gel the IPG strip was incubated in equilibration buffers I (6 M Urea, 2% SDS, 0.375 M Tris (pH 8.8), 20% Glycerol and 2% (w/v) of DTT) and II (6 M Urea, 2% SDS, 0.375 M Tris (pH 8.8), 20% Glycerol and 2.5% (w/v) of Iodoacetamide) for 20 minutes each with gentle shaking. The strip was rinsed in 1 × Tris glycine buffer (pH 8.3) and loaded along with protein molecular weight maker (Amersham Biosciences). The strip was sealed with 1% sealing agarose prepared in 1 × Tris glycine buffer (pH 8.3) along with a pinch of Bromo Phenol Blue. Following the 2-DE the gel was stained with Coomassie Blue, destained, documented and analyzed for spot pattern against duplicates. Gels were captured using Fluor-S Multi Imager (Bio-Rad), and the images were analyzed using PDQUEST software version 6.0 (Bio-Rad, USA).

### MALDI AND MS-MS analysis

#### Peptide mass finger printing (PMF)

Trypsin digested samples for PMF was reconstituted in 50% Acetonitrile (AcN) and 0.1% TFA and about 0.8 μl of it was co-crystallized on a MALDI target with 1 mg/ml of alpha-Cyano 4 hydroxycinnamic acid (CHCA) prepared in 50% AcN. Matrix Assisted Laser Desorption Ionization (MALDI) MS was performed on Voyager De STR (Perspective Biosystems) in reflector mode, with intensity of 2500.

The mass fingerprint obtained was analyzed using Data Explorer software (Applied Biosystems). The monoisotopic peaks obtained were submitted for database search. Matrix science search engine (http://www.matrixscience.com) was used to compare the peptide mass fingerprint. Carbamidomethylation of cysteine (C) and Oxidation of methionine (M) were selected as fixed and variable modifications respectively. Peptide tolerance was set to 100 ppm. Query was submitted to NCBI database. Matches with Molecular weight search (MOWSE) scores above 62 were considered as significant. The digest was dried and subjected for MS-MS analysis.

### MS/MS analysis

The digested sample was reconstituted in 5–7 μl of 2% AcN containing 0.1% Acetic acid, and desalted using Zip-Tip μ C-18 column (Millipore). The zip tips were regenerated and equilibrated sequentially with a solution of 0.1% Acetic acid in water containing 100%, 50% and 0% AcN respectively. The bound peptides were eluted with 3–5 μl of 50% AcN and 0.1% acetic acid. For ESI-MS analysis 2 μl of the sample was filled in NanoES spray capillaries of medium size (Proxeon Biosystems). The MS analysis was performed on Hybrid Quadrupole TOF mass spectrometer (API QSTAR PULSAR i, PE SCIEX) with spraying voltage of 1000 volts. A mass scan range of 500–1200 Da was used. MS-MS spectrum was acquired by collisionally induced dissociation at 40–45 eV energy using nitrogen as CAD gas. Reserpine (1 pm/μl) was used as calibration standard, and an average of 100 scans were taken for both the standard and the sample. The MS/MS spectra thus obtained was analyzed with Bioanalyst software and sequence was submitted to NCBI nr database to obtain protein identity. In the MS tag obtained Isoleucine (I) and Leucine (L) were considered to be interchangeable as these aminoacids are isobars and hence distinction on basis of mass cannot be made for these amino acids. This condition was applicable for Lysine (K) and Glutamine (Q) as well.

### Cloning and over-expression of Riken cDNA 1700026L06

Total RNA was isolated from testis tissue by Trizol method and cDNA synthesized using Gene-Amp RNA-PCR kit as per manufacturer’s protocol. The entire cDNA (1700026L06 Riken cDNA) was cloned in pET-15b (Amp^R^) (Novagen) plasmid. The gene of interest was amplified from mouse testis cDNA by PCR. Primers included the start codon and the stop codon along with restriction enzyme sites i.e., Nde1 in the forward primer (5’CGCAGCCATATGAATGAGGTGAAAG-3’), and BamH1 in the reverse primer (5’GCGCGGATCCCTATGTCGTCCCATG-3’) respectively. Protein was over-expressed in BL21 (DE3) cells using IPTG (1 mM/ml) and purified using Ni-NTA affinity column (Millipore).

### Antibody preparation

Antibody against the 1700026L06 Riken cDNA encoded protein was raised by immunizing two rabbits with *E. coli* derived recombinant full-length protein with Freund’s complete adjuvant (Bangalore Genei) for first immunization. The next three consecutive booster doses were delivered at intervals of 10 days along with Freund’s incomplete adjuvant (Bangalore Genei). The serum collected from the bleed of the immunized animal was used as antibody.

### Western blot analysis

The sperm and tissue lysates were electrophoresed on 12% SDS- polyacrylamide gel. Proteins were electro-transferred from gel onto PVDF membrane (Millipore) using standard protocol [26]. The blot was soaked in a blocking solution of 5% non-fat milk diluted in Tris buffered saline containing 0.1% Tween20 (TBST). Primary antibody of appropriate dilution in TBST was added to the blot and incubated at room temperature for one hour. The dilutions used for primary antibodies were anti-Calreticulin 1:2000 (Cell Signaling Technology 2891) anti-β-Tubulin 1:2000; (Santa Cruz Biotechnology Inc.) anti serum caldendrin 1:1000, anti-SOD 1:2000 (Santa Cruz Biotechnology Inc), anti-acrosin 1:2500 (Santa Cruz Biotechnology Inc.) and Rabbit polyclonal anti-1700026L06 Riken cDNA encoded protein 1:500. Depending upon the animal in which the primary antibody was raised appropriate HRP conjugated secondary antibody (Santa Cruz Biotechnology Inc.) diluted in TBST was added to the blot and was incubated for one hour. Blot was developed using western blotting luminol reagent (Santa Cruz Biotechnology Inc.) for Enhanced Chemi-luminiscence (ECL) as per the manufacturer’s instructions and signal was captured on X-Ray film (Kodak).

### Immunolocalization on sperm

Caudal sperms were collected and washed in Tris-buffered saline (TBS pH 7.4). Cells were fixed in 2% paraformaldehyde pH 7.2 (prepared freshly in TBS) for 10 minutes and spread on glass cover slips (Fisher Scientific, U.S.A) and air dried at 37°C. Prior to staining, cover slips were dipped in ice-cold methanol for exactly 1 minute to permeabilize the cells. Cells were treated with 0.1% Triton X-100 prepared in PBS for one hour, followed by blocking agent (5% BSA prepared in TBS containing 0.1% Triton X-100) for one hour. Cells were incubated in primary antibody diluted 1:250 in PBS containing 0.1% Triton X-100 and 1% BSA in a humid chamber for 2 hours. Washes with PBS containing 0.2% and 0.1% Triton X-100 respectively for 5 minutes each was followed by wash in PBS alone. Cells were incubated in fluorescent-labeled secondary antibody (Goat anti-rabbit diluted in PBS with 1% BSA) at 37°C, 1:300 for 1 hour. Subsequent washing was carried out in PBS containing 0.1% Triton X-100 and in PBS only for 10 minutes each. Cover slip was mounted on glass slides (Fisher Scientific, U.S.A) with antifade containing DAPI (2.5 mg/ml) as a counter stain to visualize nucleus. For the negative control the same methodology was applied except that in place of primary antibody either pre-immune serum or PBS was used.

Cells were viewed in Axioplan epifluorescence microscope and finally scanned using LSM 510 Meta Confocal Laser Scanning microscope (Carl Zeiss, Germany). The cells were optically sectioned using excitation wavelength of 488 nm under 63× objective (1.4 N.A). The emission was collected using 500–530 BP for Alexa 488. Z-sections were taken at every 0.25 μm interval. Images were projected using LSM software (Carl Zeiss, Germany) supplied with the machine.

### Immunolocalization in testis

Testes were fixed in 10% neutral buffered formalin overnight at room temperature, then processed with a standard protocol and embedded into paraffin wax. 3–4 μm sections were cut from the embedded blocks and floated onto a warm (42°C) water bath from where they were picked up onto Superfrost plus slides (Fisher Scientific). Paraffin sections were dewaxed and rehydrated by washing with xylene and ethanol, and finally in water. Sections were subjected to antigen retrieval by boiling in sodium citrate buffer for 10–15 min. Non-specific interaction was blocked by incubating in 3% bovine serum albumin (BSA) in PBS for 1 hour. Sections were incubated with antisera raised against MAST (1:200 dilution) overnight at 4°C. Fluorescent labeled Goat anti Rabbit IgG was used as secondary antibody at a dilution of 1:300 in PBS along with 1% BSA for one hour at 37°C. Finally slides were washed and mounted with antifade containing Propidium Iodide (2.5 mg/ml). For the negative control the same methodology was applied except that in place of primary antibody either pre-immune serum or PBS was used.

Tissue sections were optically sectioned with LSM 510 Meta Confocal Laser Scanning microscope (Carl Zeiss, Germany), using excitation wavelengths of 488 nm and 543 nm, laser lines (for Alexa 488 and PI respectively) at 63× objective (1.4 N.A.). The emission was collected using 500–530 BP and 565–615 BP filters for Alexa 488 and PI respectively in the multi-track mode. Z-sections were taken at every 0.38 μm interval. Images were projected using LSM software (Carl Zeiss, Germany).

### Semi-quantitative RT-PCR

RNA was isolated from mouse tissues (kidney, brain, heart, spleen, liver from both males and females, testis and ovary) using routine protocols. 1 μg of RNA from each tissue was reverse transcribed using GeneAmp RNA PCR Core kit (Life Technologies) and quantitated with GAPDH primers. Equal quantity of cDNA was PCR-amplified using primers to MAST (Forward primer 5’CGCAGCCATATGAATGAGGTGAAAG3’; reverse primer 5’GCGCGGATCCCTATGTCGTCCCATG3’). Amplification was done following a temperature profile of – denaturation at 94°C for 2 minutes, annealing at 58.6°C for 40 seconds, extension at 72°C for 1 minute; final extension of 72°C for 7 minutes. PCR products were checked on 1.5% agarose gels.

### Co-immunoprecipitation

Co-immunoprecipitation was done using Pierce cross link immunoprecipitation kit (Thermo Scientific, USA) according to manufacturer’s instruction. Briefly, the antibody was coupled to protein A/G plus agarose beads by incubation at room temperature for 1 hour. In order to prevent the co-elution of antibody with antigen, the coupled antibody was incubated with DSS (Disuccinimidyl Suberate) for half an hour. Testis lysate was pre-cleared using control agarose resin by incubating at 4°C for 1 hour. Pre cleared lysate was loaded onto column containing cross linked antibody and incubated with gentle end–over–end mixing or shaking for 1–2 hours or over night at 4°C. After washing with wash buffer, antigen was eluted using elution buffer and loaded in SDS PAGE, transferred onto nitrocellulose membrane. Blots were blocked in 0.05% Tris-buffered saline (TBS), 20% Tween and 5% non-fat milk followed by probing with the indicated primary (caldendrin anti sera, MAST antisera, anti calreticulin, anti acrosin and anti SOD) and secondary antibody. Immunoreactivity was detected with an enhanced chemi-luminiscence system.

## Competing interests

The authors declare that they have no competing interests.

## Authors’ contributions

RB identified MAST, raised antibody and did the immunolocalization and western blots. MDS raised the antibody to caldendrin and did the Co-IP studies, immunolocalization on paraffin sections and RT-PCR; VMD participated in the MS analysis, RAJ conceived and developed the project. All authors read and approved the final manuscript.

## Supplementary Material

Additional file 1: Figure S1 The MS-MS spectrum for MAST protein. Figure shows the MS-MS spectrum and MS tag obtained for MAST protein. The MS tag identified this protein as the hypothetical protein corresponding to Riken cDNA 1700026L06. Click here for file
