# Supplementary figures and images for: A mouse protein that localizes to acrosome and sperm tail is regulated by Y-chromosome
Source: BMC Cell Biol. 2013 Nov 20;14:50. doi: 10.1186/1471-2121-14-50 (PMC4225516; doi:10.1186/1471-2121-14-50)

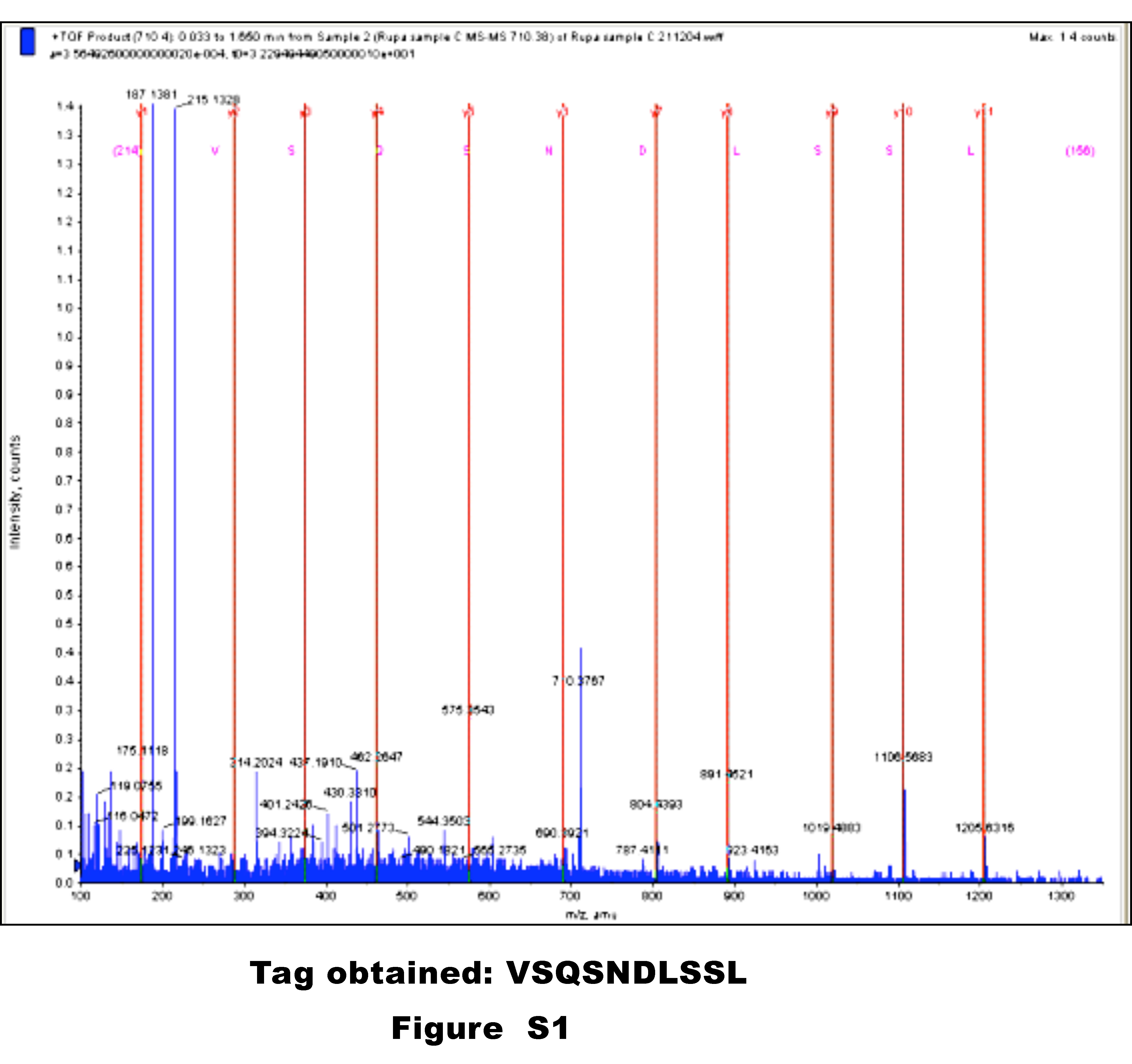

Supplement: Additional file 1: Figure S1 — The MS-MS spectrum for MAST protein. Figure shows the MS-MS spectrum and MS tag obtained for MAST protein. The MS tag identified this protein as the hypothetical protein corresponding to Riken cDNA 1700026L06. [file 1471-2121-14-50-S1.jpeg]
